# Group Hunting—A Reason for Sociality in Molossid Bats?

**DOI:** 10.1371/journal.pone.0009012

**Published:** 2010-02-03

**Authors:** Dina K. N. Dechmann, Bart Kranstauber, David Gibbs, Martin Wikelski

**Affiliations:** 1 University of Konstanz, Chair of Ornithology, Konstanz, Germany; 2 Max Planck Institute for Ornithology, Department of Migration and Immuno-Ecology, Radolfzell, Germany; 3 Department of Ecology and Evolutionary Biology, Princeton University, Princeton, New Jersey, United States of America; University of Oxford, United Kingdom

## Abstract

Many bat species live in groups, some of them in highly complex social systems, but the reasons for sociality in bats remain largely unresolved. Increased foraging efficiency through passive information transfer in species foraging for ephemeral insects has been postulated as a reason for group formation of male bats in the temperate zones. We hypothesized that benefits from group hunting might also entice tropical bats of both sexes to live in groups. Here we investigate whether *Molossus molossus*, a small insectivorous bat in Panama, hunts in groups. We use a phased antenna array setup to reduce error in telemetry bearings. Our results confirmed that simultaneously radiotracked individuals from the same colony foraged together significantly more than expected by chance. Our data are consistent with the hypothesis that many bats are social because of information transfer between foraging group members. We suggest this reason for sociality to be more widespread than currently assumed. Furthermore, benefits from group hunting may also have contributed to the evolution of group living in other animals specialized on ephemeral food sources.

## Introduction

Animals had solitary lifestyles to begin with. Sociality presumably evolved whenever group living was advantageous for the individuals in question. Many extant animals live in social groups, suggesting that benefits of group living are wide-spread. The large mammalian order of bats is one taxon representing the full range of social systems from solitary lifestyle to highly complex social systems [1]. Thus, bats offer a great opportunity to study the advantages and disadvantages of being social. Most of our knowledge about bat social systems is based on temperate zone species, where most social groups are seasonal. Seasonality in social lifestyle presumably shows that benefits of sociality outweigh the costs when a) females are reproductive and profit from communal breeding (female colonies), b) males of species that are specialized on ephemeral diet profit from improved foraging efficiency through information transfer during times of high food availability (male colonies), or c) when individuals benefit from mating aggregations (multimale-multifemale colonies: summarized in [2]. Finally, bats may aggregate without forming any social bonds, due to limited roost availability, especially in hibernacula [3]. In contrast to temperate seasonal bats, most tropical bat species are social year-round [1]. Presumably, all costs of sociality, postulated based on studies in the temperate zones, apply in tropical bats, e.g. enhanced competition for food or roosts in groups, increased parasite transmission rates or the inability to regulate body temperature individually [3], [4], [5]. The main benefit of female groups, thermoregulation during pregnancy and lactation, is presumably less expressed in tropical bats, due to high and relatively stable ambient temperatures. However, other forms of beneficial cooperation between colony members, such as allogrooming or -feeding may occur in the roost (e.g. [6], [7], in both tropical and temperate zone bats.

Social foraging, one cooperative behaviour that may occur outside the roost is displayed by a few tropical bat species, especially the spear-nosed bat, *Phyllostomus hastatus*, a frugivore, where female roost members actively recruit each other to fruiting trees with the help of individually recognizable screech calls [8]. Frugivorous bats may also use their roosts as information centres and learn about food preferences of group members from their smell [9]. This kind of flexible learning might enable individual bats to follow each other to food sources, such as fruiting trees. In contrast, highly ephemeral food sources such as insect swarms cannot be shared over repeated foraging sessions as they move unpredictably in space and time and can be dispersed by wind or rain. Information about them can only usefully be exchanged directly during an ongoing foraging flight. Eavesdropping, i. e. learning about the foraging success of group members by listening to the change in their echolocation call structure upon finding food, has been observed in several bat species [10], [11], [12], [13]. In addition, one study showed experimentally that eavesdropping might in some cases be non-opportunistic [14]. In the lesser bulldog bat, *Noctilio albiventris*, information is passively transferred via inadvertently produced cues, forcing roost members to emerge together and coordinate their spatial movements to remain within hearing distance of each other. Bats can hear and recognize each others' echolocation calls and consequently also a change in call structure, from a much larger distance than they can actively detect and localize prey [14], [15]. Call recognition is particularly important for species foraging in open space. Narrow-winged bat species that fly in open space have relatively low maximum amplitudes of echolocation frequencies, narrow frequency bands, and loud echolocation calls [16], all of which make their calls audible over large distances.

We hypothesize that an important reason for male and female tropical bats to form long term aggregations is to profit from more efficient foraging via information transfer, postulated previously as a reason for sociality in males of narrow-winged temperate-zone species [2]. Large swarms of insects in the temperate zones occur mainly during the summer, explaining why males profiting from information transfer to find this patchy but abundant resource more efficiently only from short-lived colonies. However, seasonality in the tropics is much less pronounced and insect swarms, though still ephemeral in their distribution, may occur all year. In other words, group hunting via passive information transfer might be an important strategy for tropical bats year-round and thus tightly linked to permanent sociality in bats. We predict that social foraging occurs in both sexes of narrow-winged open aerial foraging bats that live in social groups year-round and feed on an ephemeral diet and/or have a sufficiently short foraging period to make an increase in foraging efficiency through information transfer useful. In order to test this prediction, we radio-tracked groups of the extremely narrow winged aerial insectivore *Molossus molossus* during their entire nocturnal foraging periods and assessed the percentage of time spent foraging in groups. Knowing that the error in bearings gained from regular hand-telemetry can be quite large, we used a phased array antenna setup in additional to conventional Yagi-antennas to reduce directional noise in the bearing data from telemetry signals.

## Methods

### Study Site and Capture

**Figure 1:**
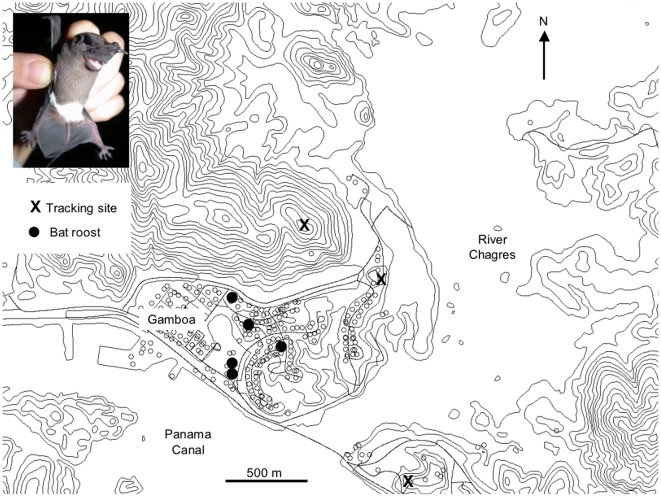
Map of the study area showing the Chagres river entering the Panama Canal and the village of Gamboa, the location of roosts where radio-tracked bats were caught as well as our main tracking points.

Our study site was the village Gamboa (N 09,07; W 079,41) in Panama, and surrounding areas, especially the Chagres River just before it enters the Panama Canal (Figure 1). The study area is covered by semi-deciduous tropical lowland rainforest with a distinct wet and dry season [17], [18]. We caught bats with mistnets when they emerged from five different daytime roosts (roosts A-E; Figure 1) in houses in Gamboa around sunset on the evenings of the 23, 26, and 30 March 2009, as well as 1 and 2 April 2009. We placed all bats in soft cloth bags upon capture and processed them as soon as evening emergence was over and no more bats were leaving the roost. We then measured and weighed bats, determined their sex and reproductive status, marked them with a subcutaneous transponder (Euro I.D., Weilerswist, Germany) and finally glued a LB-2N 0.35 g radio transmitter (Holohil, Canada) to either all (roosts A, B) or a subset of the bats from a roost (roosts C, D. E; if too many bats had been caught for simultaneous radiotracking). The average mass of individuals was 10 g, thus the transmitters remained well under the recommended 5% upper weight limit [19]. After processing we released all bats at the site of capture. Radiotracking did not start until the following evening to minimize the influence of the capture event and application of the radio transmitter on the behaviour of the bats.

### Telemetry

Three teams of two radiotrackers each with synchronized watches were stationed at elevated points around the River Chagres (Figure 1), and one additional person observed the exit hole of the bats' day roost to communicate to the rest of the team via cell phones and radios whenever one of the bats carrying radio transmitters left or returned to the roost. Due to the low signal strength and thus short reception range of the small transmitters, radio signals could not be received permanently from all tracking points along the Chagres River. Nevertheless, we continuously tracked from all vantage points as we could not predict where the bats would be foraging. To track groups of bats we used AR8200 telemetry receivers (AOR U.S.A., INC., Torrance, CA 90501) and 3-element Yagi antennae (ATS, Isanti, MN 55040). At two of the three locations we used the simple Yagi antennae to determine directions to a signal reaching a directional accuracy of <15°, which is well in the range of the expected error using a conventional setup [20], [21]. At one central station, on a canopy tower, we fixed two Yagi antennas in parallel on a wooden pole, two wavelengths apart, to form a single, phased array. The pole was then placed on a tripod to allow for simple field operations and quick directional scanning. We determined directions to a signal by lining up the antenna beam with a precise directional compass (Suunto, Finland). This simple antenna array enabled us to reliably reach directional accuracies of <3°, determined by tracking a person carrying a radio transmitter at the distance bats were foraging, in a blind experimental setup. We only included data gathered with the central phased antenna array setup in the statistical analysis.

Bearings from the same bat had to be at least two minutes apart to be included in the dataset to avoid pseudoreplication. Two minutes exceed the time bats needed to cross the entire study site. The teams of observers noted the compass bearing of the signal of each audible radio transmitter every two minutes, scanning through the frequencies of all bats as quickly as possible in a predetermined sequence. We tracked up to eight bats simultaneously, but received a signal from a maximum of four during the same 30-second interval (see below). Scanning of signals was continued until the last bat had returned to the roost or its radio signal had not been detected for 30 minutes past the time the last bat had entered the roost on the previous day. *Molosssus molossus* forages for a short period just after sunset and sometimes again for a similar time span in the morning (seeresults). As this species very efficiently removes the glued-on transmitters by scratching them off, the numbers of tracked bats in each colony decreased every night and tracking was limited to two to four evening foraging sessions and up to three morning foraging sessions per roost.

### Analysis

We compared the time spent outside the roost within and between colonies, to confirm the short activity period of this species. We give all times as means in minutes ± standard error unless otherwise indicated. The statistical tests we used are mentioned in the text, but all data analyses were done in R version 2.10 [22].

Quantification of group foraging - To quantify group foraging we took each bearing of each bat and determined which other bats' transmitter signal had been recorded within the same 30 second forward time window. We then calculated how many observations had been made within ±3° of another. To determine if bats were within ±3° of each other by chance or on purpose, we calculated a null model of co-location probability in the following way: we randomly drew the same number of bearings from all bearings per tracking session (evening or morning) and calculated how many were within a 3° angle in either direction of a random bearing. This randomization procedure was repeated 100 times. Using this bootstrapping approach we were able to compare the amount of actual group foraging with a random sample to assess if bats were found together in space and time more often than expected by chance.

Previous studies investigating group foraging had focused on either male [2] or female [14] groups. In our study, both males and females from the same groups were tracked and, in addition to quantifying group foraging over all individuals, we also compared the amount of group foraging in each sex. For this, we calculated group foraging of males and females as a fraction of all observations of each individual, provided we had more than five observations. We used a Mann-Whitney U-test to determine whether there was a difference between males and females in the tendency to forage in groups. To visualize the degree to which each colony member was involved in group foraging we drew a network graph with Netdraw 2.084 (Analytic Technologies, Lexington, KY).

**Figure 2:**
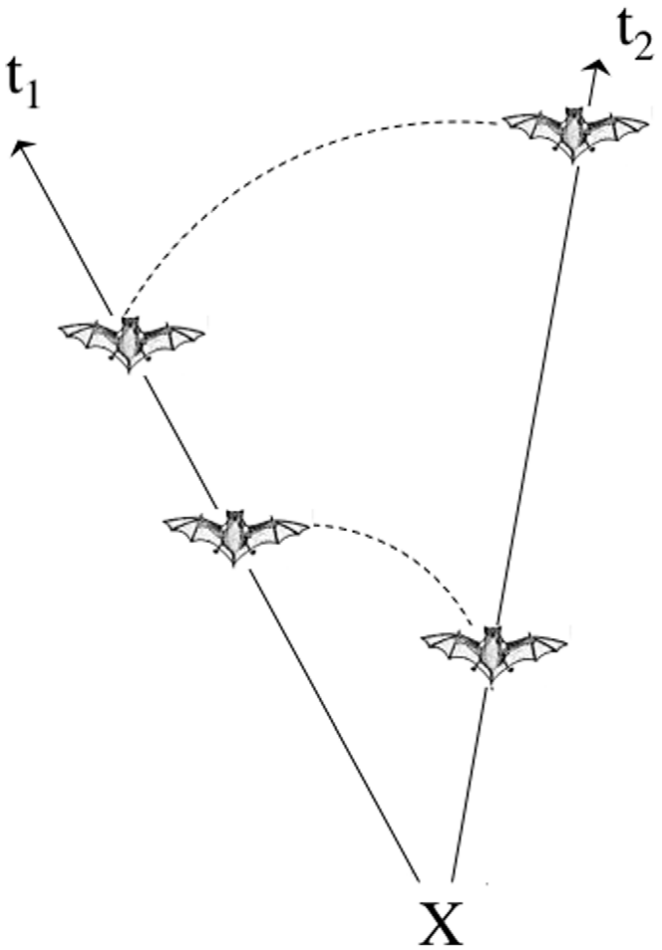
Visualisation of the complexity of a random movement that would lead to a false conclusion of coordinated movement between two bats. X: point from which bearings are taken by the observer; t1:position of the two bats along the same axis from the observer at time interval 1; t2: position of the same two bats along a different axis from the observer at time interval 2. The bats appear together (i.e. the strongest signal from their transmitter comes from the same direction) to the observer although they are not. It is very unlikely that movements like this would lead to a high percentage of co-locations in the dataset.

Coordinated movement of bats - The comparison of single bearings does not distinguish between opportunistic group foraging (i.e. each bat flies alone, but approaches successfully foraging conspecifics when it hears them) and the coordinated movement of colony members foraging together by staying within hearing distance. To test whether group members stay within hearing distance, we compared instances when the same pair of bats had been localized within 15 seconds of each other twice at an interval of 90 to 180 seconds. Finally, to show that bats did not simply remain in the same spot and thus only appeared to forage together, we also quantified which proportion of foraging pairs of bats changed bearings in synchrony between time intervals. In addition to showing coordinated movement, this analysis is a validation of our use of single bearings instead of the conventionally used cross-bearings. Theoretically, in a single bearing two apparently co-localized bats could have been on the same axis from the tracker, but not close to each other (see Figure 2 for illustration). On the other hand, to be co-localized sequentially but to have different bearings towards the same receiver would imply a complex movement that is extremely unlikely. As we frequently lost contact with transmitters we were unable to follow pairs of bats over longer time periods (which does not mean they stopped foraging together). Consequently, in our analysis we only looked at two consecutive events of group foraging.

## Results

We caught 51 individuals of *Molossus molossus* from the five roosts and randomly selected eight males and 23 females to track. Bats from the investigated colonies left their roosts for an average of 37.55±2.06 min in the evenings. The longest time a bat spent outside the roost in the evening was 83.6 min (female nr. 15 on the evening of 31. March from roost C). Only bats from the first three colonies left the roost again in the morning for a second foraging bout lasting 35.7±4.32 min, with a maximum of 92 minutes by male nr. 3 from roost A on the morning of 25. March. There was no significant difference between colonies in the amount of time spent foraging in the evening, regardless of whether they went out again in the morning. Males and females spent similar time outside the roost (5 males, 18 foraging sessions, 32.15±4.42 min; 24 females, 292 foraging sessions, 38.09±2.05 min, t-test, t = 1.21, p = 0.23). Males and females did not differ in the number of occasions where two bats were close to each other in time and space (Mann-Witney U-test, p = 0.1261).

**Figure 3:**
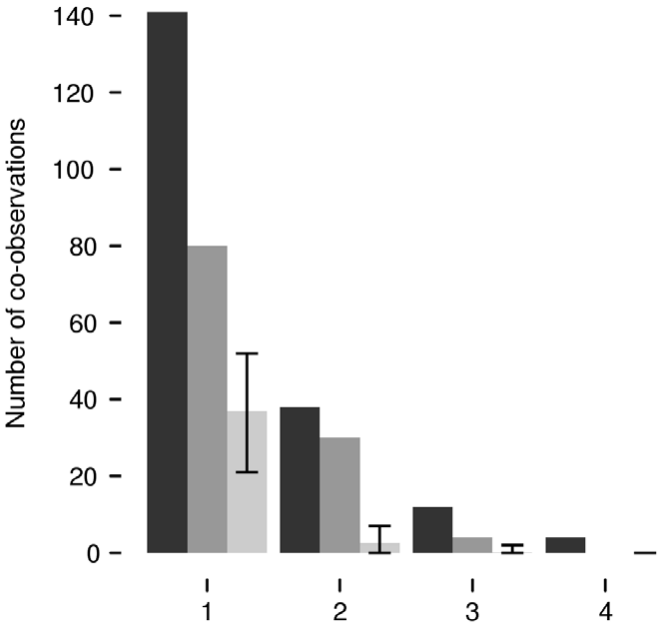
Number of other bats (1, 2, 3. or 4) actually found near a focal bat or expected by chance, summarized over all roosts and tracking sessions. First cluster of bars on x-axis: Black bar: number of occasions another bat was found in the same time window (n = 141), dark grey bar: number of occasions where this other bat also had the same compass bearing (±3°; n = 80), light grey bar: the random number of occasions another bat would be expected in the same time window and with the same compass bearing (n = 37.01 range 21 to 52). Group foraging occurred significantly more often than expected by chance. Additional clusters of bars: actual numbers of occasions that 2, 3 or 4 other bats were near in time or in time and space and the corresponding random expectations. Error bars indicate the minimum and maximum found with randomization through bootstrapping. See text for details.

Quantification of group foraging - Our dataset from the canopy tower consisted of a total of 579 independent observations of bats (i.e., radio bearings that were 2 minutes apart). We found 269 occasions where two observations had been made during the same time window. In 152 of those occasions the two observations had been close in space, i.e., 57% of observations that were made in the same time window were 6° or less apart. The null model of bearing randomization predicted an expected mean number of 42.77 (31–59) instances of bats foraging together in space and time. Bootstrapping confirmed that bats were found foraging together significantly more often than expected by chance (p<0.01; Fig. 3).

**Figure 4:**
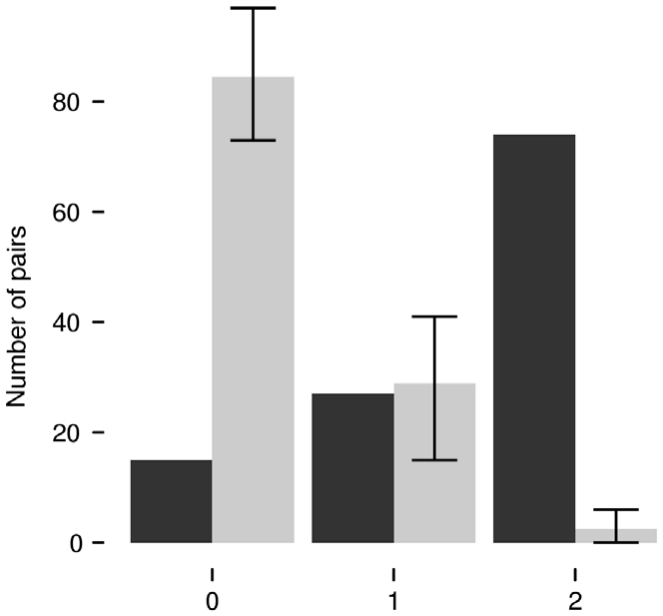
Pairs of bats that moved in a coordinated way. Showing the number of occasions when pairs of bats were co-observed in the same time window in two subsequent time intervals (time only, n = 116), where they were also found together in space one of those two occasion (time and space once, n = 27), and those where the pair of bats was together in time and space twice and thus had moved in a coordinated way (time and space twice, n = 74). Grey column: random replicates obtained with bootstrapping, black columns observed values. Error bars indicate minimum and maximum values. Coordinated movement was found more often than expected by chance.

**Figure 5:**
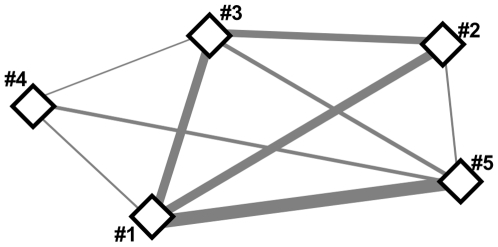
Network graph showing the example of the first *Molossus molossus* colony (roost A) we radiotracked. Thickness of lines between individuals (diamonds) illustrates the number of times these two bats were found together. Number of co-observations range from 2 (between bats nr. 3 and 4) to 18 (between bats 1 and 5). Individual nr. 2 was a male.

Coordinated movement of bats - During 116 occasions, two bats were located within the same 15 second forward time window and again within a subsequent interval of 90 to 180 seconds. In 27 of those 116 occasions, the bats were only once also within 6° of each other. However, in 74 occasions two bats were co-located within 6° of each other during both time intervals. We hypothesize that under the latter circumstances the two bats had flown together in a coordinated movement. The median difference between two subsequent bearings (90 to 180 seconds apart) was 21°, indicating that bats moved in their foraging habitat between the 2-minute scanning periods. Bootstrapping the actual bearings of the same tracking session from the same bats showed that in a total of 28.99 occasions (15–41), pairs of bats were expected to be spatially and temporally close during one of the observations. The probability of being co-located during both observations was very low; only during 2.53 occasions (0–6) bat pairs were expected to still be together by chance. We conclude that coordinated movement of two bats, indicating non-opportunistic group foraging, occurred significantly more often than expected by chance (bootstrapping as above; p<0.01; Figure 4). All bats of both sexes, except individuals 20 and 21 from roost D, and individual 28 from roost E participated in group foraging (Figure 5).

## Discussion

Many mammals including humans are social, highlighting the fact that benefits of group living often outweigh its costs. In order to investigate what benefits may have contributed to the evolution of sociality, we quantified the amount of group foraging in a social bat species by radio tracking groups of *Molossus molossus* at the same time. Our results, showing significantly more group foraging than expected by chance, confirm that increased foraging efficiency through information transfer might be an important factor promoting sociality in bats feeding on ephemeral food sources.

Social foraging is a fairly widespread phenomenon and usually involves active information transfer between individuals, examples being the honeybee dance [23] or the screech calls used by frugivorous bats to recruit group members to fruiting trees [8]. However, passive information transfer as a “byproduct” of cues inadvertently produced by foraging individuals can also yield valuable information to observing group members. Long established in birds and other socially foraging animals, passive information transfer is emerging as a potential reason for sociality in bats with an ephemeral insect diet. While birds predominantly use visual cues during passive information transfer [24], [25], [26], [27], [28], nocturnally foraging bats eavesdrop on each others inadvertently produced echolocation calls to increase the detection distance of insect prey [2], [14]. In cases of bats feeding on insect swarms, unpredictable in time and space, as well as short lived, this food source cannot be shared by recruitment of conspecifics over large distances or long time.

Sound has to travel through the air to the object, in this case the insect, and back to the sender to be perceptible as an echo, and is thus strongly subjected to attenuation. In contrast, sound only has to travel one way for bats to hear each other. Thus, bats can indirectly “detect” food over much larger distances when listening to the change in each others' echolocation calls. In *M. molossus* direct detection distance of a single 3.5–7 mm insect is estimated to be 0.5–2 m (based on the calculations used in [29], a main call frequency of 36 kHz and a source level of 113 [15]). In contrast, the distance from which this species can hear conspecific echolocation calls (i.e. the “feeding buzz” produced when a bat finds prey and attempts to capture it) under the same conditions is estimated to be 54 m. Thus, bats specialized on ephemeral insect swarms should forage within hearing distance of each other, as has been shown for female groups of *Noctilio albiventris* [14]. The overt expression of group foraging should be detectable as coordinated movement of individuals, as we were able to confirm for *M. molossus*. In fact, among bats feeding on ephemeral insects, open aerial foragers, such as *M. molossus* should profit from group hunting particularly strongly as their constant-frequency echolocation calls can travel over a long distance, increasing the area that can be covered but are not well suited for accurate prey localization. To optimize this further, bats should fly in a fanned out formation allowing them to cover a maximum area via eavesdropping and make use of the manifold increase of indirect prey detection, however, this remains to be experimentally verified. One must also keep in mind that *M. molossus* does not forage for single prey items as assumed in the detection distance estimate, but for swarms which should be detectable from a farther distance even if this would still be much less than the hearing distance.

We suggest that the main reason for an apparently low reported incidence of group foraging in bats is a methodological inadequacy of radio telemetry in small mobile animals: fast flight speeds of bats and short reception ranges of radio transmitters make group observations of bats exceedingly hard. Even in our study species, chosen because of its small foraging range in open habitat as well as its very short foraging time, we could not follow all individuals continuously. Thus, we are likely to underestimate group foraging by making a type-II error (not detecting an incidence despite its regular occurrence). Nonetheless, we found as much as 57% of group foraging, much more than expected by chance. Even the value of 57% is a very conservative estimate considering that not all bats in each group were tagged, some may have already lost their transmitters and others may simply have been out of range, but still group foraging. Further support for predominant group foraging is provided by the fact that all tracked individuals group foraged at least part of the time. An additional problem in detecting group foraging is the time it takes to scan through a multitude of radio frequencies in sequence. Once the radio frequency of a third or fourth bat is scanned, it might already have moved too far to be recognized as a foraging group member.

Our analysis of coordinated movement of bats showed that *Molossus* bats did not merely aggregate at insect swarms, thus leading us to conduct a type-I error (assuming active group foraging even though groups only aggregate by chance). Most insects have adaptations to bat predation, such as ears and behavioural responses, and insect swarms often scatter after bats start foraging in them [30]. Thus bats have to move on to the next swarm, but are unlikely to move together by chance. Such non-random, or coordinated movements are further supported by our bootstrapping methods suggesting that bats had similar compass bearings more often than by chance. Furthermore we suggest that bats move together over large spatial scales, because the median change in bearings between consecutive localizations of pairs was 21° (much more than the 6° of our error).

An important question we were unable to address here is whether there is an optimal group size, and if so, what group number is optimal under given conditions [31]. We could not track all animals at all times, thus we were limited to investigating whether group foraging is taking place at all. Therefore we focussed on pairs of bats in our analysis and are unable to quantify how large the actual foraging groups are. However, there were many instances where we found up to four bats spatially and temporally close to each other in up to 5 consecutive time intervals (Figure 3), indicating that larger groups than pairs indeed forage together, also during long time periods.

Eavesdropping in bats has been observed in a variety of species and may be very widespread, but it is probably most frequently opportunistic, meaning that bats on the wing hear echolocation calls produced during prey capture and feeding (i.e. feeding buzzes) of another con- or heterospecific bat and approach the source of the sound to profit from the same food source. However, the emerging picture from recent studies including the one we present here, is different: particularly bats feeding on ephemeral insect swarms may forage socially, and to do so emerge from the roost together and keep flying together during foraging trips. Of particular interest in *M. molossus* and other molossid bats is that they produce “social calls” (i.e. calls at frequencies below 18–20 kHz) in addition to the echolocation calls while foraging (personal observation; [11], [32]) the role of which remains completely uninvestigated. Our studies are only the first steps and quantification of costs and benefits in order to establish group foraging as a more general pattern is necessary. However, we hypothesize that at least in some tropical species including *Molossus molossus*, benefits from group foraging may have been an important driving force for the evolution of stable social groups.
